# Draft Genome Sequence of Pseudomonas koreensis Strain AB36, Isolated from Gold Mining Soil

**DOI:** 10.1128/MRA.00175-19

**Published:** 2019-05-16

**Authors:** Olubukola Oluranti Babalola, Ayansina Segun Ayangbenro

**Affiliations:** a Food Security and Safety Niche, Faculty of Natural and Agricultural Sciences, North-West University, Mmabatho, South Africa; University of Delaware

## Abstract

Here, we report the draft genome sequence of Pseudomonas koreensis strain AB36, isolated from gold mining soil in South Africa. The draft sequence consists of 5,902,614 bp, with a G+C content of 60.1% and 5,242 protein-coding genes. The genome provides insight into its metabolism and the degradation of environmental pollutants.

## Abstract (precis)

Here, we report the draft genome sequence of Pseudomonas koreensis strain AB36, isolated from gold mining soil in South Africa. The draft sequence consists of 5,902,614 bp, with a G+C content of 60.1% and 5,242 protein-coding genes.

## ANNOUNCEMENT

One of the most diverse bacterial genera is *Pseudomonas*. These bacterial strains have varied metabolic properties and are widely distributed in the ecosystem (1). The genus has been divided into two intrageneric groups (IGs) (IG P. aeruginosa and IG P. fluorescens) based on multilocus sequence analysis of the 16S rRNA, *gyrB*, *rpoB*, and *rpoD* genes (2). IG P. fluorescens is divided into six groups which are represented by the species P. fluorescens, P. syringae, P. lutea, P. putida, P. anguilliseptica, and P. straminea. Pseudomonas koreensis belongs to the P. fluorescens subdivision (2, 3).

Pseudomonas koreensis strain AB36 was isolated from gold-contaminated soil samples, as described by Babalola et al. (4). The genomic DNA of strain AB36 was extracted from pure culture grown on LB agar using a ZR soil microbe miniprep DNA extraction kit (Zymo Research, CA, USA), according to the kit’s protocol. The DNA was quantified with a NanoDrop Lite spectrophotometer (Thermo Fisher Scientific, CA, USA) and checked for quality on a 1% agarose gel stained with ethidium bromide. The genome of strain AB36 was sequenced on an Illumina MiSeq sequencer at Molecular Research (MR DNA), Shallowater, TX. The library was constructed using a Nextera DNA sample preparation kit (Illumina), following the manufacturer's instructions. After the library preparation, the final concentration of the library was measured using the Qubit double-stranded DNA (dsDNA) high-sensitivity (HS) assay kit (Life Technologies, Inc.). The average library size was determined using the Agilent 2100 Bioanalyzer (Agilent Technologies). The library was pooled and diluted to 12.0 pM and paired-end sequenced using a 600-cycle v.3 reagent kit (Illumina), with an average coverage of 50×.

The read quality of the raw sequence was assessed with FastQC (v.1.0.4) (5), and the reads were trimmed to remove adapter sequences, low-quality sequences, and ambiguous reads using Trimmomatic (v.0.36) (6), with default settings. *De novo* assembly was performed using SPAdes (v.3.12.0) (7), with default parameters, to yield 44 contigs. The assembled genome of strain AB36 generates a total size of 5,902,614 bp, with a G+C content of 60.1%. The contigs have N_50_ and L_50_ values of 419,669 bp and 5, respectively. The contigs were annotated with the Rapid Annotations using Subsystems Technology (RAST; v.2.0) server (8) and the NCBI Prokaryotic Genome Annotation Pipeline (PGAP; v.4.7) (9), which identified 5,242 coding genes, 71 RNA genes, 60 tRNA genes, and 159 pseudogenes.

The annotated draft genome showed the presence of genes responsible for metal resistance, such as arsenic resistance protein (ArsH), cobalt-zinc-cadmium resistance protein (CzcD), and chromate transport protein (ChrA). The annotated genome also carries genes for aromatic hydrocarbon (benzoate, phenylalkanoic acid, quinate, and heterocyclic aromatic hydrocarbon) degradation. The biosynthetic gene cluster (BGC) analysis was carried out using antiSMASH (v.3.0) (10), and seven BGCs were predicted. Most BGCs are predicted to be nonribosomal peptide synthetase clusters, which include known clusters such as pyoverdine, jagaricin, and mangotoxin clusters. Other BGCs are for aryl polyene, bacteriocin, and beta-lactone. The draft genome of *P. koreensis* provides a wealth of information about the aromatic hydrocarbon degradative genes and metal resistance genes that are of environmental importance.

### Data availability.

This whole-genome shotgun project has been deposited at DDBJ/ENA/GenBank under the accession number SEUB00000000. The version described in this paper is the first version, SEUB01000000. The Sequence Read Archive (SRA) accession number is SRR8529805.
